# Supplementary material for: Structural elucidation of a novel mechanism for the bacteriophage-based inhibition of the RNA degradosome
Source: eLife. 2016 Jul 22;5:e16413. doi: 10.7554/eLife.16413 (PMC4980113; doi:10.7554/eLife.16413)
Supplement: Supplementary file 1. — Table 1. MS results of the affinity purifications on Rne::StrepII, infected with one of seven Pseudomonas phages. Table 2. Diffraction statistics and refinement statistics of the crystals of Dip. DOI: http://dx.doi.org/10.7554/eLife.16413.023 [file elife-16413-supp1.docx]

**Table 1. MS results of the affinity purifications on Rne::StrepII, infected with one of seven Pseudomonas phages.**

The numbers indicate the ‘Total spectral Count’ identified for a specific protein. Proteins with an asterisk were also purified during affinity purifications using other bacterial complexes and are considered as false positives (Van den Bossche et al., 2014).

| Protein | gene | PA-number | gi-number | Accession number | Mass (Da) | Control^+^ (10 min) | 14/1^+^  (5 min) | φKZ^+^  (15 min) | LUZ19^+^ (5 min) | LKA1^+^  (10 min) | LUZ24^+^ (15 min) | PEV2^+^ (10 min) | YuA+  (25 min) |
| --- | --- | --- | --- | --- | --- | --- | --- | --- | --- | --- | --- | --- | --- |
| φKZ _gp37 | ORF37 |  | gi\|29134973 | NP_803603 | 30,944.20 |  |  | 38 |  |  |  |  |  |
| 14-1_gp70* | ORF69 |  | gi\|218148610 | YP_002364378 | 29,231.50 |  | 5 |  |  |  |  |  |  |
| PEV2_gp43 (conserved homologue LIT1_gp43)* | ORF43 |  | gi\|282598890 | YP_003358440 | 63,543.70 |  |  |  |  |  |  |  | 3 |
| YuA_ gp66* | ORF66 |  | gi\|162135148 | YP_001595889 | 54,013.40 |  |  |  |  |  |  | 1 |  |
|  |  |  |  |  |  |  |  |  |  |  |  |  |  |
| ribonuclease E | *rne* | PA2976 | gi\|15598172 | NP_251666 | 117,464.80 | 129 | 360 | 182 | 154 | 245 | 364 | 83 | 55 |
| polynucleotide phosphorylase/polyadenylase | *pnp* | PA4740 | gi\|15599934 | NP_253428 | 75,454.20 | 44 | 388 | 45 | 301 | 285 | 365 | 166 | 60 |
| ATP-dependent RNA helicase | *rhlE* | PA2840 | gi\|15598036 | NP_251530 | 62,109.20 |  | 23 | 35 | 1 | 1 | 15 |  |  |
| ATP-dependent RNA helicase | *deaD* | PA0428 | gi\|15595625 | NP_249119 | 70,112.70 | 10 | 22 |  |  | 3 | 7 |  | 2 |
| ATP-dependent RNA helicase RhlB | *rhl* | PA3861 | gi\|161486761 | NP_252550 | 44,288.60 |  | 9 |  |  | 1 | 12 |  |  |
| RNA-binding protein Hfq | *hfq* | PA4944 | gi\|15600137 | NP_253631 | 9,103.50 |  |  |  |  | 3 |  |  |  |
|  |  |  |  |  |  |  |  |  |  |  |  |  |  |
| (3R)-hydroxymyristoyl-ACP dehydratase | *fabZ* | PA3645 | gi\|15598841 | NP_252335 | 16,774.30 |  |  |  |  | 1 |  |  |  |
| ABC transporter ATP-binding protein |  | PA4595 | gi\|15599791 | NP_253285 | 61,304.10 |  |  | 2 |  |  |  |  |  |
| acetyl-CoA carboxylase biotin carboxyl carrier protein subunit | *accB* | PA4847 | gi\|15600040 | NP_253534 | 16,454.70 | 7 | 18 | 27 | 57 | 26 | 28 | 2 |  |
| alginate regulatory protein AlgP | *algP* | PA5253 | gi\|15600446 | NP_253940 | 34,492.00 |  | 5 |  |  | 7 |  |  |  |
| Anaerobically-induced outer membrane porin OprE precursor | *oprE* | PA0291 | gi\|15595488 | NP_248982 | 49,668.90 |  |  |  | 1 |  | 2 |  |  |
| branched-chain alpha-keto acid dehydrogenase subunit E2 | *bkdB* | PA2249 | gi\|15597445 | NP_250939 | 45,755.10 |  |  | 1 |  |  |  |  |  |
| DNA-binding protein HU | *hupB* | PA1804 | gi\|15597001 | NP_250495 | 9,086.90 | 7 |  | 7 | 8 | 15 |  | 2 | 1 |
| DNA-directed RNA polymerase subunit alpha | *rpoA* | PA4238 | gi\|15599434 | NP_252928 | 36,650.50 |  |  | 6 |  | 1 | 1 |  |  |
| DNA-directed RNA polymerase subunit beta | *rpoB* | PA4270 | gi\|15599466 | NP_252960 | 150,841.60 |  |  |  | 1 |  |  |  |  |
| DNA-directed RNA polymerase subunit beta' | *rpoC* | PA4269 | gi\|15599465 | NP_252959 | 154,368.60 |  | 3 | 1 |  | 2 |  |  |  |
| elongation factor Tu | *tufA* | PA4265 | gi\|15599461 | NP_252955 | 43,369.40 | 6 | 3 | 5 |  | 2 | 1 | 3 |  |
| F0F1 ATP synthase subunit alpha | *atpA* | PA5556 | gi\|15600749 | NP_254243 | 55,394.20 | 4 |  |  |  |  |  |  |  |
| F0F1 ATP synthase subunit B | *atpF* | PA5558 | gi\|15600751 | NP_254245 | 16,956.60 |  | 2 |  |  |  |  |  |  |
| F0F1 ATP synthase subunit beta | *atpD* | PA5554 | gi\|15600747 | NP_254241 | 49,500.40 | 3 | 4 | 5 |  | 2 | 4 | 6 |  |
| GTP-binding protein EngA |  | PA3799 | gi\|15598994 | NP_252488 | 55,007.10 |  |  |  |  |  | 3 |  |  |
| hypothetical protein PA3179 |  | PA3179 | gi\|15598375 | NP_251869 | 43,724.60 |  | 1 |  |  |  |  |  |  |
| hypothetical protein PA4460 |  | PA4460 | gi\|15599656 | NP_253150 | 19,107.60 |  |  | 1 |  |  |  |  |  |
| hypothetical protein PA4753 |  | PA4753 | gi\|15599947 | NP_253441 | 11,640.10 |  |  | 2 |  |  |  |  |  |
| lysozyme inhibitor | *mliC* | PA0867 | gi\|15596064 | NP_249558 | 13,695.50 |  | 3 |  |  | 1 |  |  |  |
| Major porin and structural outer membrane porin OprF precursor | *oprF* | PA1777 | gi\|15596974 | NP_250468 | 37,639.00 | 1 | 15 | 5 | 6 | 4 | 8 | 6 | 6 |
| molecular chaperone DnaK | *dnaK* | PA4761 | gi\|15599955 | NP_253449 | 68,403.60 |  |  |  |  |  |  | 3 |  |
| motility regulator | *morA* | PA4601 | gi\|15599797 | NP_253291 | 159,669.70 |  | 2 |  |  |  |  |  |  |
| Outer membrane lipoprotein OprI precursor | *oprI* | PA2853 | gi\|15598049 | NP_251543 | 8,835.10 |  |  |  | 3 | 4 |  |  |  |
| Outer membrane protein OprG precursor | *oprG* | PA4067 | gi\|15599262 | NP_252756 | 25,194.60 |  |  |  |  |  | 3 |  |  |
| Peptidoglycan associated lipoprotein OprL precursor | *oprL* | PA0973 | gi\|15596170 | NP_249664 | 17,925.10 |  | 4 |  |  | 3 | 2 |  |  |
| peptidyl-prolyl cis-trans isomerase, FkbP-type |  | PA3262 | gi\|15598458 | NP_251952 | 26,846.20 |  |  |  |  |  | 1 |  |  |
| PhoP/Q and low Mg2+ inducible outer membrane protein H1 precursor | *oprH* | PA1178 | gi\|15596375 | NP_249869 | 21,575.30 |  | 2 | 3 | 1 |  | 3 |  |  |
| poly(A) polymerase | *pcnB* | PA4727 | gi\|15599921 | NP_253415 | 53,302.70 |  | 1 |  |  |  | 1 |  |  |
| polyhydroxyalkanoate synthesis protein PhaF | *phaF* | PA5060 | gi\|15600253 | NP_253747 | 30,578.90 |  | 1 | 1 | 4 | 12 |  |  | 2 |
| preprotein translocase subunit SecD | *secD* | PA3821 | gi\|15599016 | NP_252510 | 67,677.00 |  | 5 |  |  |  |  |  |  |
| preprotein translocase subunit YajC |  | PA3822 | gi\|15599017 | NP_252511 | 11,862.10 |  |  |  |  | 1 |  |  |  |
| recombinase A | *recA* | PA3617 | gi\|15598813 | NP_252307 | 36,879.80 |  |  |  |  |  | 2 |  |  |
| signal recognition particle protein Ffh | *ffh* | PA3746 | gi\|15598941 | NP_252435 | 49,361.00 |  |  | 2 | 5 | 5 | 2 | 1 |  |
| transcription termination factor Rho | *rho* | PA5239 | gi\|15600432 | NP_253926 | 47,071.70 |  | 2 |  |  |  |  |  |  |
| transcriptional regulator MvaT, P16 subunit | *mvaT* | PA4315 | gi\|15599511 | NP_253005 | 14,181.10 |  |  |  |  | 2 |  |  |  |
| translation initiation factor IF-2 | *infB* | PA4744 | gi\|15599938 | NP_253432 | 90,911.00 |  |  |  | 3 | 5 |  |  | 3 |
| translation initiation factor IF-3 | *infC* | PA2743 | gi\|15597939 | NP_251433 | 20,882.70 |  |  | 2 | 10 | 15 | 8 | 2 | 1 |
| type 4 fimbrial precursor PilA | *pilA* | PA4525 | gi\|15599721 | NP_253215 | 15,512.20 |  | 2 |  |  |  | 2 |  |  |
| 30S ribosomal protein S1 | *rpsA* | PA3162 | gi\|15598358 | NP_251852 | 61,869.90 | 16 | 5 | 27 |  |  | 24 |  | 3 |
| 30S ribosomal protein S2 | *rpsB* | PA3656 | gi\|15598852 | NP_252346 | 27,337.30 |  |  | 3 | 2 |  | 13 |  |  |
| 30S ribosomal protein S3 | *rpsC* | PA4257 | gi\|15599453 | NP_252947 | 25,838.40 | 11 | 9 | 18 | 12 | 11 | 2 |  |  |
| 30S ribosomal protein S4 | *rpsD* | PA4239 | gi\|15599435 | NP_252929 | 23,277.90 |  | 3 |  |  |  | 1 |  |  |
| 30S ribosomal protein S5 | *rpsE* | PA4246 | gi\|15599442 | NP_252936 | 17,625.00 |  | 13 | 8 | 1 | 3 | 6 | 2 | 1 |
| 30S ribosomal protein S6 | *rpsF* | PA4935 | gi\|15600128 | NP_253622 | 16,164.50 |  | 1 |  |  | 2 | 3 |  |  |
| 30S ribosomal protein S7 | *rpsG* | PA4267 | gi\|15599463 | NP_252957 | 17,504.70 |  | 7 |  |  | 1 | 2 |  | 3 |
| 30S ribosomal protein S9 | *rpsI* | PA4432 | gi\|15599628 | NP_253122 | 14,597.00 |  | 6 | 2 |  | 1 | 2 |  |  |
| 30S ribosomal protein S10 | *rpsJ* | PA4264 | gi\|15599460 | NP_252954 | 11,766.70 |  |  |  |  | 2 |  |  |  |
| 30S ribosomal protein S11 | *rpsK* | PA4240 | gi\|15599436 | NP_252930 | 13,629.70 | 4 | 3 | 3 | 1 | 5 | 12 |  | 1 |
| 30S ribosomal protein S12 | *rpsL* | PA4268 | gi\|15599464 | NP_252958 | 13,798.80 |  | 4 |  |  |  |  |  |  |
| 30S ribosomal protein S13 | *rpsM* | PA4241 | gi\|15599437 | NP_252931 | 13,266.00 |  |  |  |  | 1 |  |  |  |
| 30S ribosomal protein S15 | *rpsO* | PA4741 | gi\|15599935 | NP_253429 | 10,098.00 |  |  | 4 |  | 5.00 |  |  |  |
| 30S ribosomal protein S16 | *rpsP* | PA3745 | gi\|15598940 | NP_252434 | 9,204.50 | 1 |  | 5 | 8 | 8 | 1 | 2 |  |
| 30S ribosomal protein S18 | *rpsR* | PA4934 | gi\|15600127 | NP_253621 | 8,873.90 |  |  |  |  | 2 |  |  |  |
| 30S ribosomal protein S19 | *rpsS* | PA4259 | gi\|15599455 | NP_252949 | 10,357.30 |  |  |  |  | 2 |  |  |  |
| 30S ribosomal protein S20 | *rpsT* | PA4563 | gi\|15599759 | NP_253253 | 9,918.00 |  |  |  |  | 4 | 2 |  |  |
| 30S ribosomal protein S21 | *rpsU* | PA0579 | gi\|15595776 | NP_249270 | 8,484.90 |  |  |  |  | 3 |  |  |  |
| 50S ribosomal protein L1 | *rplA* | PA4273 | gi\|15599469 | NP_252963 | 24,234.00 | 7 | 19 | 8 | 17 | 10 | 2 | 3 |  |
| 50S ribosomal protein L2 | *rplB* | PA4260 | gi\|15599456 | NP_252950 | 29,579.30 |  | 19 | 1 | 1 | 1 | 1 |  |  |
| 50S ribosomal protein L3 | *rplC* | PA4263 | gi\|15599459 | NP_252953 | 22,590.60 |  |  | 7 | 1 | 13 |  |  |  |
| 50S ribosomal protein L4 | *rplD* | PA4262 | gi\|15599458 | NP_252952 | 21,639.90 | 3 | 5 | 1 | 8 | 16 | 5 |  |  |
| 50S ribosomal protein L5 | *rplE* | PA4251 | gi\|15599447 | NP_252941 | 20,393.20 |  | 12 |  |  | 4 | 10 |  |  |
| 50S ribosomal protein L6 | *rplF* | PA4248 | gi\|15599444 | NP_252938 | 19,099.00 |  |  |  |  | 3 |  |  |  |
| 50S ribosomal protein L10 | *rplJ* | PA4272 | gi\|15599468 | NP_252962 | 17,634.50 |  | 12 |  | 2 | 7 | 6 |  |  |
| 50S ribosomal protein L11 | *rplK* | PA4274 | gi\|15599470 | NP_252964 | 14,907.30 |  | 5 |  | 1 | 2 | 2 |  |  |
| 50S ribosomal protein L13 | *rplM* | PA4433 | gi\|15599629 | NP_253123 | 16,028.70 |  | 3 | 1 |  | 3 | 2 |  |  |
| 50S ribosomal protein L14 | *rplN* | PA4253 | gi\|15599449 | NP_252943 | 13,411.90 |  |  |  |  | 2 |  |  |  |
| 50S ribosomal protein L15 | *rplO* | PA4244 | gi\|15599440 | NP_252934 | 15,174.60 |  | 12 | 1 | 2 | 16 | 4 |  |  |
| 50S ribosomal protein L16 | *rplP* | PA4256 | gi\|15599452 | NP_252946 | 15,401.50 |  | 8 | 1 | 3 | 3 | 3 |  |  |
| 50S ribosomal protein L17 | *rplQ* | PA4237 | gi\|15599433 | NP_252927 | 14,504.30 |  | 2 |  |  |  |  |  |  |
| 50S ribosomal protein L19 | *rplS* | PA3742 | gi\|15598937 | NP_252431 | 13,032.40 |  | 3 | 3 | 3 | 14 | 5 |  |  |
| 50S ribosomal protein L20 | *rplT* | PA2741 | gi\|15597937 | NP_251431 | 13,365.70 |  | 6 |  | 1 | 1 |  |  |  |
| 50S ribosomal protein L21 | *rplU* | PA4568 | gi\|15599764 | NP_253258 | 11,635.20 | 3 | 12 | 4 |  | 7 | 5 |  | 2 |
| 50S ribosomal protein L22 | *rplV* | PA4258 | gi\|15599454 | NP_252948 | 11,911.10 |  |  |  |  | 2 |  |  |  |
| 50S ribosomal protein L23 | *rplW* | PA4261 | gi\|15599457 | NP_252951 | 10,949.90 |  |  |  |  | 4 | 1 |  |  |
| 50S ribosomal protein L27 | *rpmA* | PA4567 | gi\|15599763 | NP_253257 | 8,990.40 |  |  |  |  | 2 |  |  |  |
| 50S ribosomal protein L28 | *rpmB* | PA5316 | gi\|15600509 | NP_254003 | 9,065.60 |  |  |  |  | 2 |  |  |  |
| 50S ribosomal protein L29 | *rpmC* | PA4255 | gi\|15599451 | NP_252945 | 7,201.50 |  |  |  |  | 8 |  |  |  |
| 50S ribosomal protein L30 | *rpmD* | PA4245 | gi\|15599441 | NP_252935 | 6,477.60 |  |  |  |  | 2 |  |  |  |
| 50S ribosomal protein L36 | *rpmJ* | PA4242 | gi\|15599438 | NP_252932 | 4,434.30 |  |  |  |  | 2 |  |  |  |

**Table 2. Diffraction statistics and refinement statistics of the crystals of Dip.**

|  | Dip- SeMet | Dip:RNase E756-775 Native |
| --- | --- | --- |
| Diffraction Statistics |  |  |
|  |  |  |
| Space group | P21 | P1 |
| Cell dimensions |  |  |
| a, b, c (Å) | 72.0, 65.0, 85.4 | 84.6, 84.6, 84.6 |
| α, β, γ (°) | 90.0, 107.7, 90.0 | 107.9, 107.9, 107.9 |
| Resolution | 68.6-2.20 (2.27-2.20) | 72.2 -2.75 (2.84-2.75) |
| Rmerge | 0.11 (0.81) | 0.03 (0.43) |
| CC(1/2) | 0.99 (0.53) | 0.99 (0.59) |
| I/σI | 8.2 (2.1) | 12.3 (1.7) |
| Completeness (%) | 99.8 (99.9) | 94.2 (93.2) |
| Anomalous completeness (%) | 97.0 (97.3) | - |
| Multiplicity | 5.2 (5.3) | 1.9 (1.8) |
| Number of unique reflections | 38321 | 39553 |
| Wilson B-factor | 42.0 | 98.7 |
|  |  |  |
| Refinement statistics |  |  |
|  |  |  |
| R work | 0.19 | 0.26 |
| R free | 0.22 | 0.29 |
| Number of atoms | 4310 | 11570 |
| Protein | 4060 | 11569 |
| water | 250 | 1 |
| r.m.s deviations |  |  |
| Bond lengths (Å) | 0.004 | 0.005 |
| Bond angles (°) | 0.66 | 0.74 |
| Ramachandran favoured (%) | 97.2 | 92.3 |
| Ramachandran allowed (%) | 2.6 | 6.5 |
| Ramachandran outliers (%) | 0.2 | 1.2 |
|  |  |  |
